# Inter-individual variation in genes governing human hippocampal progenitor differentiation *in vitro* is associated with hippocampal volume in adulthood

**DOI:** 10.1038/s41598-017-15042-z

**Published:** 2017-11-08

**Authors:** Timothy R. Powell, Tytus Murphy, Sang H. Lee, Rodrigo R. R. Duarte, Hyun Ah Lee, Demelza Smeeth, Jack Price, Gerome Breen, Sandrine Thuret

**Affiliations:** 1 King’s College London, Social, Genetic and Developmental Psychiatry, Institute of Psychiatry, Psychology and Neuroscience, London, United Kingdom; 2 National Institute for Health Research, Biomedical Research Centre for Mental Health, Institute of Psychiatry, Psychology and Neuroscience at the Maudsley Hospital and King’s College London, London, United Kingdom; 3 King’s College London, Department of Basic and Clinical Neuroscience, Institute of Psychiatry, Psychology and Neuroscience, London, United Kingdom

## Abstract

Hippocampal volumes are smaller in psychiatric disorder patients and lower levels of hippocampal neurogenesis are the hypothesized cause. Understanding which molecular processes regulate hippocampal progenitor differentiation might aid in the identification of novel drug targets that can promote larger hippocampal volumes. Here we use a unique human cell line to assay genome-wide expression changes when hippocampal progenitor cells differentiate. RNA was extracted from proliferating cells versus differentiated neural cells and applied to Illumina Human HT-12 v4 Expression BeadChips. Linear regressions were used to determine the effect of differentiation on probe expression and we assessed enrichment for gene ontology (GO) terms. Genetic pathway analysis (MAGMA) was used to evaluate the relationship between hippocampal progenitor cell differentiation and adult hippocampal volume, using results from the imaging genomics consortium, ENIGMA. Downregulated transcripts were enriched for mitotic processes and upregulated transcripts were enriched for cell differentiation. Upregulated (differentiation) transcripts specifically, were also predictive of adult hippocampal volume; with Early growth response protein 2 identified as a hub transcription factor within the top GO term, and a potential drug target. Our results suggest that genes governing differentiation, rather than mitosis, have an impact on adult hippocampal volume and that these genes represent important drug targets.

## Introduction

The specialisation of pluripotent stem cells into neurons, commonly referred to as “neurogenesis”, occurs across all brain regions during foetal development^1^. Different niches of neural stem cells give rise to specialised neurons, glial cells and oligodendrocytes which inhabit specific brain regions^2^. Neurogenesis is affected by genetic, environmental, hormonal and epigenetic factors^3–5^, and these likely moderate or trigger a series of molecular changes, resulting in the termination of a neural stem cell’s proliferation and the initiation of cell differentiation.

The hippocampus is a brain structure important in learning, memory and mood, and interestingly continues to exhibit neurogenesis throughout an individual’s lifetime^6–10^. Smaller hippocampal volumes have been linked to a variety of psychiatric disease states including post-traumatic stress disorder and major depressive disorder^11–13^. There are many cellular changes that could contribute to volume loss, including loss of dendritic length or spines, decreased glial size or number^14^, yet a recent rodent study demonstrated that inhibition of hippocampal neurogenesis leads to hippocampal volume reduction^15^. Therefore, a greater understanding of what molecular processes regulate hippocampal neurogenesis may prove useful in understanding the development of this brain structure, its involvement in psychiatric disease, and in the development of pharmacotherapies which aim to target and reverse smaller hippocampal volumes.

Previous work in non-human animal *in vivo* and *in vitro* systems have revealed substantial gene expression changes associated with neuronal differentiation, with Notch^16^, Wnt^17–19^ and brain derived neurotrophic factor (BDNF)^20,21^ signalling pathways hypothesized to be key regulators. However, regional specialisation in the brain is associated with specific and developmentally-sensitive changes to gene expression profiles^22^. Thus, it’s unclear whether the pathways previously implicated for neuronal differentiation are also important in regulating early hippocampal neural stem cell differentiation. Furthermore, it’s unclear what other transcriptional mechanisms might be important in regulating neural stem cell differentiation in the hippocampus in humans, in a hypothesis-free manner.

Here, we investigated genome-wide expression changes occurring when proliferating human hippocampal progenitor cells differentiate over a 7-day period. We further created a “neural progenitor differentiation gene set”, and determined which biological processes are downregulated and upregulated in response to neural progenitor differentiation. We subsequently used gene co-expression analysis to understand which hub genes are important in driving neural progenitor differentiation, and we performed genetic pathway analysis to test whether genes within our neural progenitor differentiation gene set predicted adult hippocampal volume in the ENIGMA neuroimaging genetics dataset^23^. The ultimate aim was to identify druggable genes and/or gene networks capable of promoting neural progenitor differentiation and larger hippocampal volumes.

## Methods

### The Hippocampal Progenitor Cell Line

The multipotent, human hippocampal progenitor cell line HPC0A07/03 C (provided by ReNeuron, Surrey, UK) was used for all experiments, as described previously^24–26^. ReNeuron’s HPC0A07/03 C cells were obtained from a 12-week old foetus (with a typical karyotype) and immortalised with c-mycER technology. In the presence of growth factors (FGF2 and EGF) and 4-Hydroxy-Tamoxifen (4-OHT), progenitors cells will proliferate and remain undifferentiated. Removal of these growth factors induces differentiation of cells, on average, into 52% of TuJ1-positive cells (of which 35% are doublecortin-positive neuroblasts, 25% were MAP2-positive mature neurons, and 8% labelled positive for both, doublecortin and MAP2), 27% S100ß-positive astrocytes, 2% of O1-positive oligodendrocytes and 19% of GFAP-positive immature progenitor cells^24^. Cells were grown in Dulbecco’s Modified Eagle’s Media/F12 (DMEM:F12, Invitrogen, Paisley, UK) supplemented with 0.03% human albumin (Baxter Healthcare, Compton, UK), 100 µg/ml human apo-transferrin (Sigma, St-Louis, MO, USA), 16.2 µg/ml human putrescine DiHCl (Sigma), 5 µg/ml human insulin (Sigma), 60ng/ml progesterone (Sigma), 2 mM L-glutamine (Sigma) and 40 ng/ml sodium selenite (Sigma). To maintain proliferation, 10 ng/ml human bFGF (Pepro Tech EC Ltd, London, UK), 20 ng/ml human EGF (Pepro Tech EC Ltd) and 100 nM 4-OHT (Sigma) were added to proliferating media, and removed in differentiating media. All cells were grown at 37 °C, 5% CO_2_, and in a humidified atmosphere.

### Cells used in this study and summary of cell protocol

**Figure 1:**
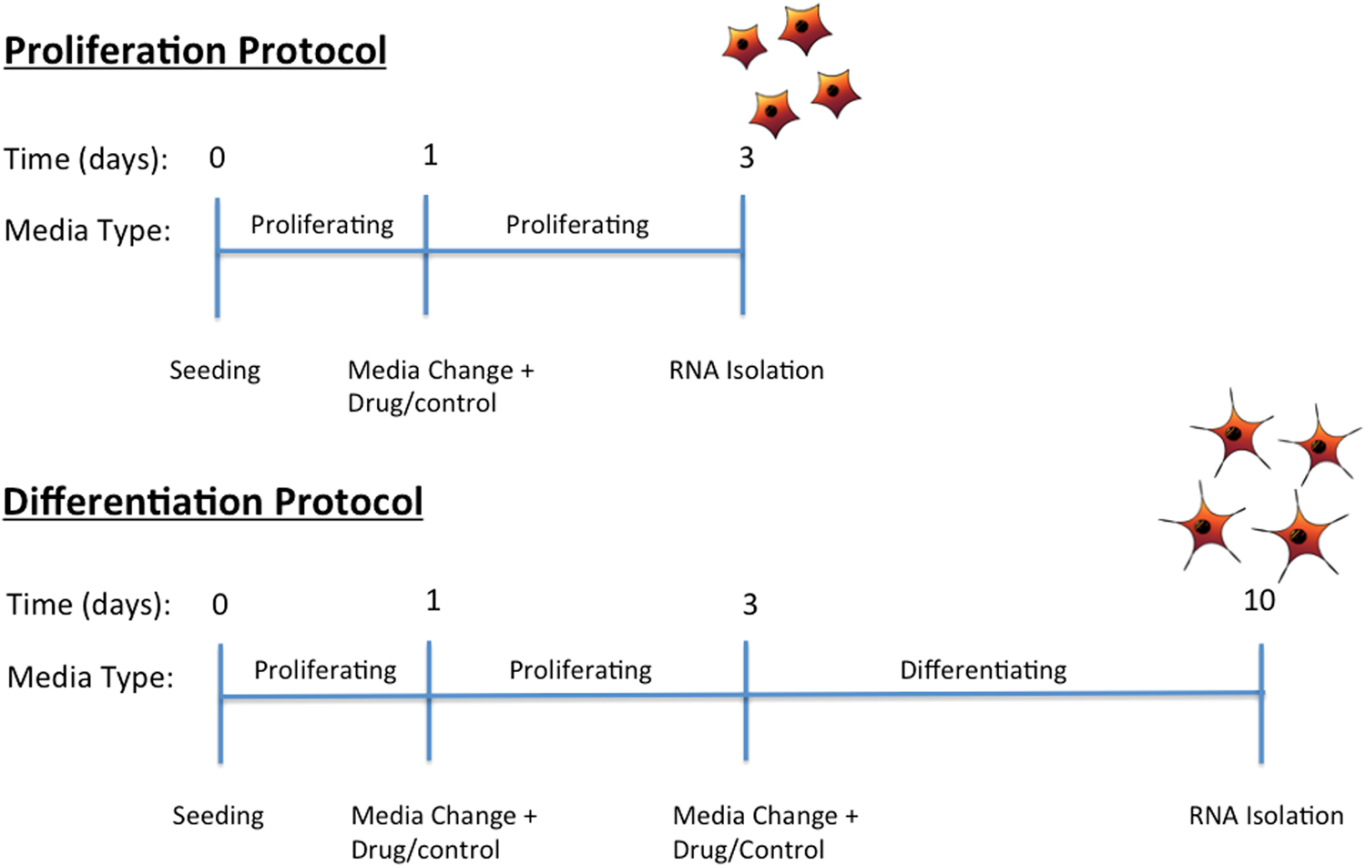
A summary of the protocol used for the proliferation and differentiation of human hippocampal progenitor cells.

Data utilised here was collected as part of a set of 24 experiments, which assayed the effects of two antidepressant drugs on both proliferating and differentiating human hippocampal progenitor cell populations (25–26; S1, Supplementary Information). Each experiment consisted of a control, low, medium and high drug dose. In the context of all adequately detected (unfiltered) probes on the microarray, neither drug had significant effects on expression of individual genes^25,26^. The inclusion of untreated control conditions allowed us to regress out any minor effects of drug and drug dose within our conservative statistical models, in order to accurately compare proliferating versus differentiating cell gene expression profiles (see below). A summary of the protocol used throughout all experiments is summarised in Fig. 1.

### Protocol for Proliferating Cells

Cells were seeded for 24 hours on laminin-coated 6 well plates (Nunclon, Roskilde, Denmark) in proliferating media. After seeding, media was changed and cells were incubated with proliferating media for a further 48 hours. Media was then aspirated and 1 ml of Trireagent (Sigma, St-Louis, MO, U.S.) was added for later RNA isolation. In total there were 48 independent proliferating cell populations grown in separate wells, which were obtained from six different passages.

### Protocol for Differentiating Cells

The differentiation protocol incorporated the same procedures as described in the proliferation protocol. However, at the end of the 48-hour proliferating conditions, media was aspirated and replaced with differentiating media (lacking EGF, bFGF, 4-OHT) twice over one hour to ensure thorough removal of growth factors. The cells were then grown in differentiating media for 7 days and differentiated into a glial/neuronal mixed culture containing a majority of neurons as shown previously^24^, confirmed here using immunocytoochemistry for S100β-positive astrocytes, doublecortin-positive neuroblasts, MAP2-positive and Prox1-positive neurons (Supplementary Information, S2), where Prox1 has been shown to be a marker for dentate gyrus neurons^27^. Subsequently, cells underwent RNA collection as above. In total there were 48 independent differentiating cell populations grown in separate wells, which were obtained from seven different passages. The majority of proliferating and differentiating cells were obtained from the same passage numbers.

### Whole Genome Transcriptomics

RNA from cell experiments was isolated using Trireagent (Sigma) following the standard protocol, with an additional ethanol precipitation step to increase RNA purity. All samples had 260/280 ratios of between 1.9 and 2.1 as tested using the Nanodrop D1000 (Thermoscientific, Wilmington, DE). All RNA samples had RNA integrity numbers (RINs) of greater than 9, as assessed using the Agilent Bioanalyzer 2100 (Agilent Technologies, California, U.S.). 300 ng of RNA from each sample was processed on Illumina Human HT-12 v4 Expression BeadChip (Illumina Inc., California, U.S.) according to manufacturer’s protocol. Initial quality control assessment was performed in Genome Studio (Illumina), where outliers were identified using a scatterplot of average signal intensities. One sample was revealed as an outlier, and this was confirmed using hierarchical clustering. This sample was subsequently removed from downstream analysis. The Lumi (Bioconductor) package in R (http://www.R-project.org) was used for quality control, quantile-normalization, log-transformation and gene annotation^28^. Genes were then filtered based on detection values generated by Genome Studio. Expression probes had to reach the detection p-value threshold <0.01 in at least one sample, and if not, they were excluded. Microarray data generated from this experiment has been made publically available on the Gene Expression Omnibus (GEO; accession number GSE95791).

### Quantitative Polymerase Chain Reaction (qPCR) Validation

Complementary DNA (cDNA) was synthesized from 500 ng of RNA and utilised for qPCR validation, as part of a three-step process. In the first step genomic DNA (gDNA) was removed using the Precision DNase kit (Primer Design, Southampton, UK), following the standard manufacturer protocol. In the second step we converted RNA to cDNA using the Reverse Transcription premix kit (Primer Design), following the standard manufacturer protocol. Finally, we performed quantitative PCR reactions in 384-well plates. All samples under investigation were tested using three technical replicates, and three no-template controls were included for each gene being investigated. An eight point human leukocyte gDNA dilution series (0.47 ng, 0.94 ng, 1.88 ng, 3.75 ng, 7.5 ng, 15 ng, 30 ng, 60 ng) was included for every gene, to create a standard curve, which was used to perform absolute quantification of each cDNA sample under investigation. Human primary cell derived BioBank T-cell cDNA (Primer Design) was also run on every plate as a positive control.

Per reaction, the qPCR mix consisted of: 10 uL Precision PLUS SYBR green Mastermix (Primer Design), 10 uL RNase free water, 200 nM forward primer, 200 nM reverse primer and 25 ng of cDNA. The thermocycling conditions consisted of three stages: Step 1: 2 minutes at 95 °C; Step 2: 40 cycles at 95 °C for 10 seconds followed by 60 °C for 1 minute (data collection); Step 3: Melting curve.

We selected the following genes for validation: brevican (*BCAN*), top upregulated transcript; Transforming Growth Factor Beta Induced (*TGFBI*), top downregulated transcript; Doublecortin (*DCX*), a neuronal-specific marker; and Cell Division Cycle 20 (*CDC20*), a cell cycle marker featured in our top downregulated gene network. For primer sequences, see Supplementary information, S3.

For validation we used eight cDNA samples derived from proliferating cells and eight cDNA samples derived from differentiating cells. We selected Vimentin (*VIM*) as an endogenous reference gene, based on our microarray results demonstrating it was well expressed with a very low level of variation across all samples (C.V. <1%). This was especially necessary as traditional housekeeping genes showed high levels of variation (C.V. >1%), including β-actin (*ACTB*) and Glyceraldehyde 3-phosphate dehydrogenase (*GAPDH)*, which showed significant changes during differentiation (*ACTB*: log2(fold change) = −0.426917281, p = 1.91E-17; GAPDH: log2(fold change) = −0.408308201, p = 6.34E-20).

Primers were designed by inputting the microarray probe sequence into BLAT (https://genome.ucsc.edu/cgi-bin/hgBlatoptio; build hg19) and identifying the exact genomic region it assays; selecting a ~400 BP region falling within a single exon covering this region; and inputting the genomic sequence into Primer3 (http://primer3.ut.ee; product range of between 80-100 BP). Each primer was designed to generate a single amplicon, and to fall within a single exon, so that it would create the same product size for either gDNA or cDNA, allowing for the accurate use of a gDNA standard curve.

### Statistical Analysis

**(i) Determining Expression Changes During Hippocampal Neural Progenitor Differentiation:** To assess the gene expression changes triggered when proliferating cells are left to differentiate, we performed a linear regression with probe expression as the dependent variable, proliferating/differentiating state as the independent variable, and array batch and biological replicate as factors. As we utilised some cell populations which had been drug treated, we also regressed out any minor influences of drug and drug dose.

**(ii) Gene Set Derivation & Gene Ontology Enrichment Of Upregulated And Downregulated Genes:** To understand which biological mechanisms may be affected by cell differentiation we first identified a “neural progenitor differentiation gene set” consisting of probes which showed both a Bonferroni-significant change in expression and a log2(fold change) >± 0.5.

To understand which biological mechanisms are being upregulated and downregulated within our neural progenitor differentiation gene set, we separately entered genes showing an increase in expression, and genes showing a decrease in expression, into the Gene Ontology enRIchment anaLysis and visuaLizAtion tool (GOrilla; http://cbl-gorilla.cs.technion.ac.il; ref.^29^), and included all probes surviving background correction as our reference list. As part of GOrilla, we tested for Gene Ontology (GO) term enrichment for Biological Processes (GOTERM_BP_FAT), Cellular Components (GOTERM_CC_FAT) and Molecular Functions (GOTERM_MF_FAT), and we used the false discovery rate (q < 0.05) to correct for multiple testing. To visualise the top GO terms, we entered genes contributing to the top upregulated and downregulated GO terms into GeneMania^30^. GeneMania uses previously published works to draw connections between genes within a user-defined list and establishes images of co-expression networks.

**(iii) Cell-Type-Associated Expression Changes During Differentiation:** To determine which cell types were most likely driving changes to either upregulated or downregulated transcripts within our neural progenitor differentiation gene set, we linked immunocytochemistry data from the three main cell types in our multipotent culture (doublecortin-, MAP2- and S100β-positive cells) to our gene expression data (S.4, Supplementary Information). Similarly to whole blood experiments, we used the variance in cell type counts between samples to tease apart the contribution each cell type had to the overall transcriptomic profile e.g. ref.^31^. In this analysis we included all 48 proliferating cell populations and assigned 0% marker expression for doublecortin, MAP2 and S100β, as cells are kept in an immature progenitor state and do not express these markers. We further included 17 differentiating cell populations for which we had percentage expression of these three markers. We subsequently performed the same linear model as in (i) in our downregulated/upregulated transcripts separately, but we also included percentage of cells expressing doublecortin, MAP2 and S100β in our model in order to tease apart cell-type-associated expression changes during differentiation. As our upregulated and downregulated gene sets had already been preselected from our main analysis (Bonferroni-significant with log2(fold change) >± 0.5), and because we had reduced power in this subset due to sample size, we applied a liberal significance threshold (p < 0.05) when determining which cell type was most likely driving expression changes during differentiation. We further determined overlapping transcripts from our cell-type-associated results, with those transcripts overexpressed in either neurons or astrocytes (relative to other central nervous system cells) from mouse brain, as described by Cahoy and colleagues^32^ (S.5–S.8, Supplementary Information).

**(iv) Genetic Pathway Analysis:** Genetic pathway analysis was used to determine the relationship between genes associated with neural progenitor differentiation (derived in (ii)) and adult hippocampal volume. As part of a sensitivity analysis, we investigated whether upregulated or downregulated genes within our neural progenitor differentiation gene set, showed stronger associative enrichment for single nucleotide polymorphisms (SNPs) nominally associated with hippocampal volume.

To achieve this, we used the pathway analysis tool, MAGMA^33^. MAGMA tests for gene set enrichment by first generating a gene-wide statistic from the GWAS results files, adjusting for gene size, single nucleotide polymorphism (SNP) density and linkage disequilibrium effects^33^. It then performs a competitive test of gene set association^33^. The competitive test of association compares how well a gene set performs relative to other gene sets of similar size across the genome.

We used a 35 kb 5′ and 10 kb 3′ window around genes to test if our neural progenitor differentiation gene set showed associative enrichment for SNPs nominally predicting hippocampal volume. GWAS summary statistics relating to hippocampal volume were made available via the ENIGMA Consortium. ENIGMA has performed the largest association studies to-date on brain volumes (meta-analyses), involving results from 50 separate studies^23^. In their hippocampal volume GWAS, they used data collected from 13,163 subjects (http://enigma.ini.usc.edu; see ref.^23^); the results of which we utilise here.

**(v) qPCR Validation:** qPCR data was output from the ABI Prism SDS Software version 2.2 which generated cycle threshold (C_*t*_) values for each sample. For a sample to be included in downstream data analysis, at least two of the C_*t*_ technical triplicates needed to achieve a standard deviation of less than 0.5. Remaining C_*t*_ values were then related to absolute quantities as part of a standard curve, creating C_*q*_ values. The mean C_*q*_ value for each target gene was divided by the mean C_*q*_ of the endogenous reference gene in order to generate relative expression. Independent t-tests were used to compare expression differences between proliferating and differentiating cells.

## Results

### Microarray analysis shows widespread transcriptional reprogramming during neural progenitor differentiation

**Figure 2:**
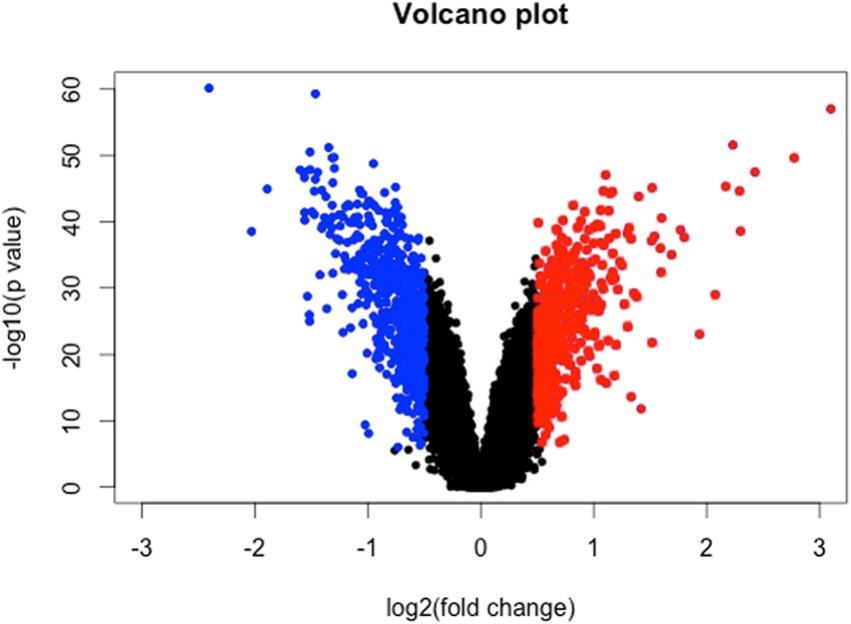
A volcano plot showing the distribution of log2(fold change) values and associated −log(p) values for 29,313 probes, where we compared expression at each probe when cells were proliferating or undergoing neural progenitor differentiation. The data points shown in blue/red represent those probes included in our neural progenitor differentiation gene set, i.e. surpassing the Bonferroni-significance threshold (p < 1.706E-06) with a log2(fold change) >± 0.5.

Our data revealed that 29,313 probes were adequately detected after background correction. Of these, 6,713 probes surpassed a Bonferroni p-value threshold of 1.706E-06. 1,141 probes were both Bonferroni significant and demonstrated a log2(fold change) of greater than ± 0.5; 652 of these transcripts were downregulated and 489 were upregulated. The distribution of p-values and log2(fold changes) are shown in Fig. 2. For full results of the genes affected during differentiation, see S.10, Supplementary Information. For results on the relationship between cell-type and expression changes, see S.5–S.8, S.11, Supplementary Information.

**Figure 3:**
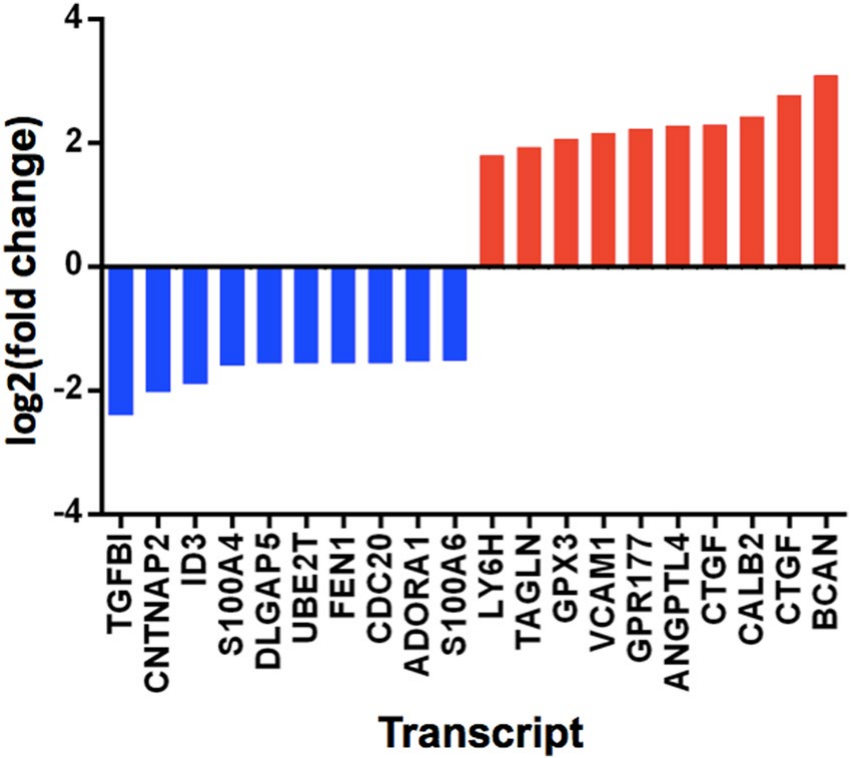
A bar chart showing the mean log2(fold change), y-axis, for the 10 transcripts showing the largest downregulation in expression (blue) and the 10 transcripts showing the largest upregulation in expression (red) when human hippocampal neural progenitor cells differentiate, x-axis. All transcripts surpassed the Bonferroni correction for multiple testing. [*TGFBI* = Transforming Growth Factor Beta Induced; *CNTNAP2* = Contactin-associated protein-like 2; *ID3* = Inhibitor Of DNA Binding 3; *S100A4* = S100 calcium-binding protein A4; *DLGAP5* = Discs Large Homolog Associated Protein 5; *UBE2T* = Ubiquitin Conjugating Enzyme E2 T; *FEN1* = Flap endonuclease 1; *CDC20* = Cell-division cycle protein 20; *ADORA1* = Adenosine A1 Receptor; *S100A6* = S100 Calcium Binding Protein A6; *LY6H* = Lymphocyte antigen 6 complex, locus H; *TAGLN* = Transgelin; *GPX3* = Glutathione peroxidase 3; *VCAM1* = Vascular cell adhesion molecule 1; *GPR117* = Wntless Wnt ligand secretion mediator; *ANGPTL4* = Angiopoietin-like 4; *CTGF* = Connective tissue growth factor; *CALB2* = Calbindin 2; *BCAN* = Brevican].

The top ten genes showing a downregulation in expression, and upregulation in expression during neural progenitor differentiation are shown in Fig. 3.

**Figure 4:**
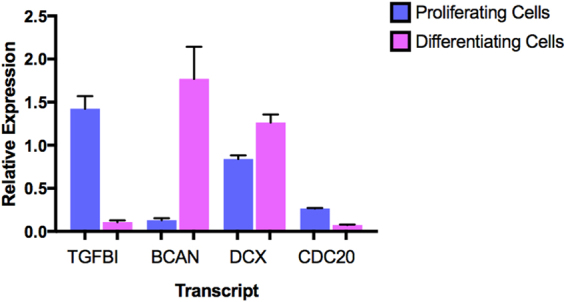
This figure shows results from our qPCR validation experiments. The bar chart shows the mean relative expression of each gene in proliferating (blue; n = 8) and differentiating (pink; n = 8) human hippocampal progenitor cells. The error bars represent the standard error of the mean. All transcripts showed significant differences between proliferating and differentiating cell populations (p < 0.005). Note: Expression of *DCX* and *CDC20* have been multiplied by a factor of 4 to increase visibility.

### qPCR Validates Microarray Findings

All standard curves showed an R^2^ ≥ 0.99 between known DNA quantity and C_*t*_ values. None of the no-template controls showed amplification for any of our genes, and all positive cDNA controls showed amplification. Dissociation curves (melting curves) revealed a clear single peak for each gene, confirming amplification specificity. All samples passed quality control checks, and demonstrated S.D. <0.5 between technical replicates. Independent t-tests confirmed significant differences between proliferating and differentiating cell populations expressing *TGFBI* (t = 9.038, d.f. = 7.338, p = 3.100E-05), *BCAN* (t = −4.390, d.f. = 7.049, p = 3.140E-03), *DCX* (t = 4.044, d.f. = 14, p = 0.001) and *CDC20* (t = 19.136, d.f. = 14, p = 1.95E-11).

### Gene Ontology analyses show a downregulation in genes controlling mitotic processes and an upregulation of genes controlling cellular differentiation

**Figure 5:**
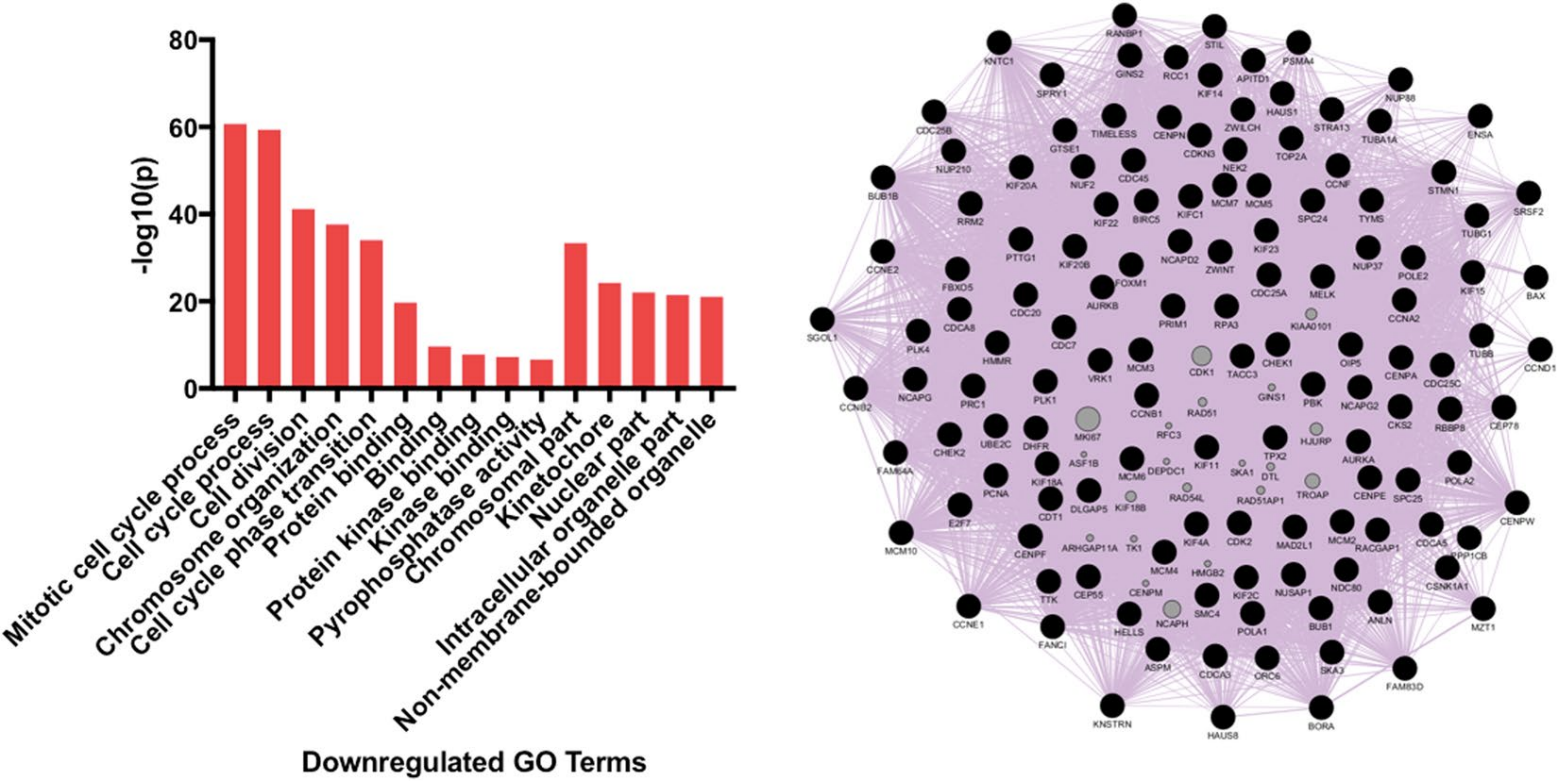
Left: The five most significantly downregulated biological processes, molecular functions and cellular components (Gene Ontology terms, from left to right) after hippocampal neural progenitor cells differentiate for 7-days. Right: Transcripts within the GO term ‘Mitotic Cell Cycle Process’ (most significant term) visualised as a gene co-expression network.

**Figure 6:**
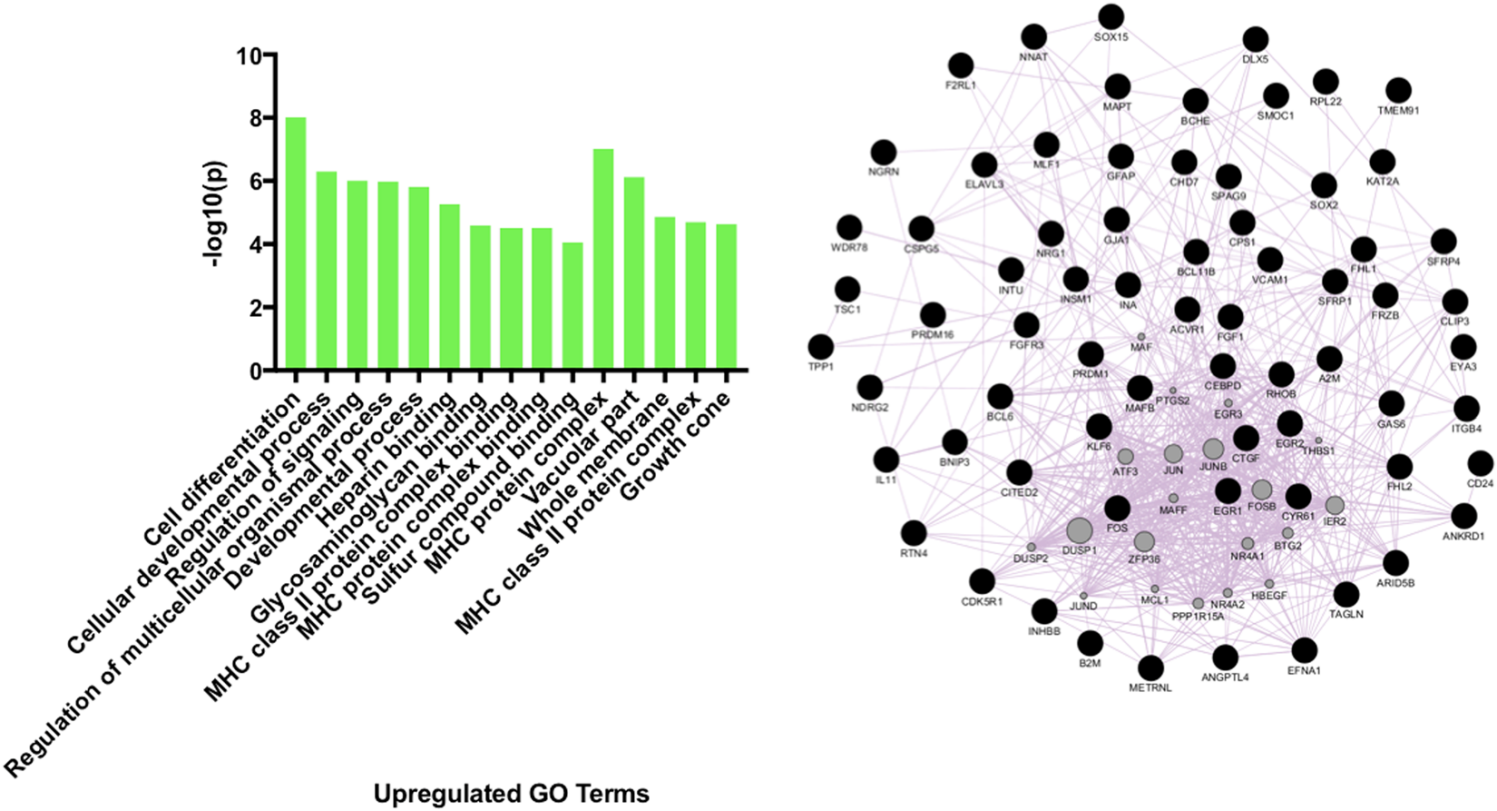
Left: The five most significantly upregulated biological processes, molecular functions and cellular components (Gene Ontology terms, from left to right) after hippocampal progenitor cells differentiate for 7-days. Right: Transcripts within the GO term ‘Cell Differentiation’ (most significant term) visualised as a gene co-expression network.

Amongst the downregulated genes, GOrilla revealed a significant enrichment for 286 biological processes, 54 molecular functions and 69 cellular components. Amongst the upregulated genes, GOrilla revealed a significant enrichment for 56 biological processes, 2 molecular functions and 27 cellular components (S.12, Supplementary Information). The five most significant of each of these GO subtypes amongst the downregulated and upregulated gene sets are shown in Figs 5 and 6, alongside the most significant GO term visualised as a gene network.

### Genetic pathway analysis reveals that upregulated genes associated with differentiation predict adult hippocampal volume

**Figure 7:**
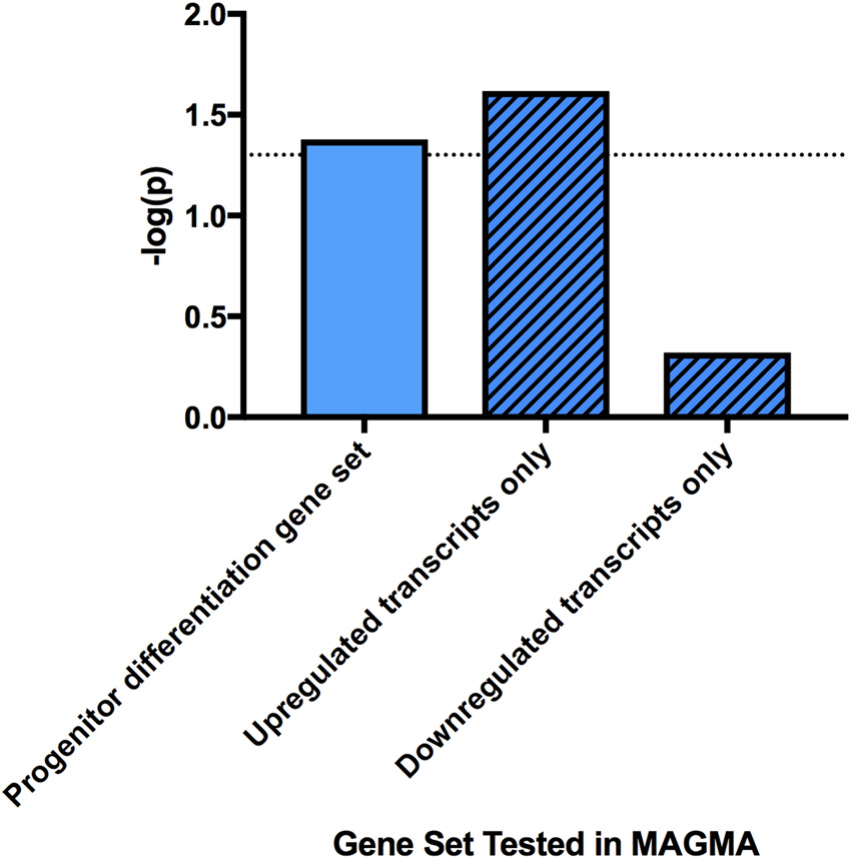
Bar charts showing results from MAGMA. Results from our competitive analyses investigating if our gene sets are enriched for SNPs predictive of adult hippocampal volume and whether they ‘outcompete’ gene sets of similar size. The initial association between our neural progenitor differentiation gene set and hippocampal volume was significant (p < 0.05). Further consideration of downregulated (mitotic processes) and upregulated (differentiation) gene sets revealed that the association is being driven by upregulated genes governing differentiation, not the downregulated genes governing mitosis. Gene sets are shown on the x-axis, and the y-axis shows the -log10(p-value) output from MAGMA. The dashed line represents a p-value threshold of p = 0.05.

The MAGMA results revealed that our neural progenitor differentiation gene set was significantly enriched for genes predictive of hippocampal volume (p = 0.031). By dissecting this gene list by upregulated/downregulated genes only, we found that it was specifically upregulated transcripts (i.e. those involved in differentiation) that were associated with hippocampal volume (p = 0.030), not downregulated transcripts (p = 0.478), Fig. 7.

## Discussion

This study is the first to investigate the genome-wide expression changes which occur during human hippocampal progenitor cell differentiation as part of a 7-day protocol, Fig. 1. Our results revealed extensive gene expression changes associated with differentiation, Fig. 2. Top expression changes included functionally relevant genes, such as Transforming Growth Factor, Beta-Induced (*TGFBI*), Figs 3–4, which codes for a protein that increases in response to TGFB^34^. TGFB has previously been identified as a negative regulator of adult hippocampal neurogenesis^35^, therefore its downregulation during progenitor differentiation is consistent with this finding. Likewise, Inhibitor Of DNA Binding 3 (*ID3*) expression has been found to inhibit neurogenesis, therefore a downregulation during differentiation^36^, Fig. 3, may act to disinhibit this mechanism. In contrast, some transcripts show an upregulation in expression during neural progenitor differentiation, such as brevican (*BCAN*), Figs 3–4, and Connective Tissue Growth Factor (*CTGF*), Fig. 3. BCAN is proteoglycan that is specifically expressed in the central nervous system and has previously been linked to neuronal differentiation^37^, and CTGF has been identified as a factor involved in regulating neuron survival^38^.

On a gene network level we found a very strong downregulation of transcripts controlling mitosis, which corresponds to the slowing down of cellular proliferation in our model, Fig. 5. Whereas, there was a simultaneous upregulation of genes governing cellular differentiation and development, corresponding to the generation of young neurons and astrocytes observed in this cell line, Fig. 6. Unlike previous studies^16–21^, we did not identify Notch, Wnt and BDNF signalling pathways, as the top regulatory pathways, as indicated by our GO terms (Figs 5 and 6). However, individual members of the Notch (*NOTCH2*, *DLL1*) and Wnt (*WNT5b*, *DVL3*) pathways were significantly affected by neuronal differentiation, and there was a significant upregulation of *BDNF*, but the fold changes associated with these were below our threshold for inclusion in our neural progenitor differentiation gene set (see S.10, Supplementary information). The partial lack of replication here may relate to the multipotent nature of the stem cell line or the fact we are primarily generating immature neurons, rather than mature ones. However, when accounting for multipotency using staining data, genes associated with either young or more mature neurons still did not include members of these pathways, which may suggest that on a genome-wide scale, and/or in human cells, they may be of less relevance to the differentiation of hippocampal progenitor cells.

By performing genetic pathway analysis, we determined a positive associative enrichment for genes predicting hippocampal volume within our hippocampal neural progenitor differentiation gene set. This suggests that we have identified a set of relevant genes affecting both neural progenitor differentiation and long-term hippocampal volume, and subsequently we may have honed in on important, novel drug targets for the treatment of neuropsychiatric conditions, where there is a smaller hippocampus^11–13^. Through dissecting our gene set by upregulated/downregulated transcripts only, we found that only genes important in differentiation (upregulated transcripts) predicted hippocampal volume, not those governing neural progenitor proliferation (downregulated transcripts), Fig. 7.

Hub genes within the top co-expression network in our upregulated gene set includes three notable transcription factors, Early growth response proteins 1 (*EGR1*) and 2 (*EGR2*) and CCAAT/enhancer-binding protein delta (*CEBPD*; Fig. 6); with all three genes implicated in both the differentiation of young doublecortin-positive neurons and astrocytes, based on our cell-type associated analyses (Supplementary information). Transcription factors, such as these ones, play a role in regulating the expression of many other genes, allowing for co-ordinated and timely changes to protein expression, required for growth, development and response to environmental stimuli^39^. Thus, the ones we identify here, probably play a pivotal role in coordinating progenitor differentiation.

EGR-1 is a transcription factor highly expressed in young doublecortin-positive cells, and it has been used as a marker of neuronal activity and circuit integration^40^. Enhanced hippocampal neurogenesis via calorie restriction has been associated with rises in EGR-1, and EGR-1 is vital for hippocampal dependent long-term memory in mice^41^. Research has shown that CEBPD is also a transcription factor important in hippocampal neurogenesis in mice^42^. CEPBP knockout mice show a reduction in the number of new-born cells in dentate gyrus of the hippocampus and a decreased number of cells differentiating into neurons^42^. Out of the three transcription factors, however, *EGR-2*, was the one with the greatest number of single nucleotide polymorphisms associated with hippocampal volume (most significant individual SNP was rs7913336, p = 0.00086; S.9, Supplementary Information), and is the gene which had the strongest change within our differentiation assay (log fold change = 1.30, p = 6.23E-25). This arguably suggests that EGR-2 may be the transcription factor of greatest importance in both coordinating neural progenitor differentiation and subsequently long-term hippocampal volume. EGR-2 has also been found to be upregulated in mouse neurons relative to other central nervous system cell types;^32^ S.5, Supplementary information. Previous research has also implicated EGR-2 in both the differentiation and myelination of nerve cells in mice^43^, and mutations within the EGR-2 gene have been associated with peripheral neuropathies, including Charcot-Marie-Tooth type 1, Dejerine-Sottas syndrome and congenital hypomyelinating neuropathy^44^. Further work is now needed to understand if modulating EGR-2 levels has a role in affecting hippocampal volume via neurogenic mechanisms.

Although our study provides potentially important insights into the cellular and molecular regulation of long-term hippocampal volume, there are several limitations to consider. First, we utilised a conditionally immortalised cell line from a single human donor. Ideally, future studies should validate findings reported here, in cells which are not artificially affected by immortalisation and are collected from several independent donors. Secondly, even though the ENIGMA GWAS on hippocampal volume involves the largest cohort to-date, it may still be underpowered, which may dilute the genetic signal and weaken the accuracy of our genetic pathway results.

In conclusion, our study is the first to describe genome-wide expression changes during human hippocampal progenitor differentiation. We provide the first empirical evidence showing a biological relationship between early hippocampal neurogenesis and long-term adult hippocampal volume in humans. These results suggest that inter-individual variation in genes triggering the differentiation of progenitor cells, evoke long lasting differences to hippocampal volume. Whereas, inter-individual variation in genes governing progenitor proliferation may be less important in affecting long-term hippocampal volume in adults. Our results differ from previous studies as we did not identify Wnt, Notch or BDNF as the most important mediators of progenitor differentiation, rather our work implicates the importance of the transcription factors *EGR1*, *CEBPD* and particularly *EGR2*. We believe the data generated within our study provides a resource for others to test for further biological associations, and our data also suggests novel targets for drugs which aim to increase hippocampal volume in the treatment of psychiatric disease.

## Electronic supplementary material

Supplementary Information

Dataset S10

Dataset S11

Dataset S12
