# Supplementary material for: Inter-individual variation in genes governing human hippocampal progenitor differentiation in vitro is associated with hippocampal volume in adulthood
Source: Sci Rep. 2017 Nov 8;7:15112. doi: 10.1038/s41598-017-15042-z (PMC5678432; doi:10.1038/s41598-017-15042-z)
Supplement: Supplementary file 1 — Supplementary Information [file 41598_2017_15042_MOESM1_ESM.doc]

Inter-individual variation in genes governing human hippocampal progenitor differentiation in vitro is associated with hippocampal volume in adulthood

Timothy R Powell^1,2^, PhD; Tytus Murphy^3^, PhD; Sang H Lee^1,2^, MSc; Rodrigo R.R. Duarte^3^, MSc; Hyun Ah Lee^3^, MSc; Demelza Smeeth^3^, MSc; Jack Price^3^, PhD; Gerome Breen^1,2*^PhD; Sandrine Thuret^3*♯^, PhD.

*Gerome Breen and Sandrine Thuret contributed equally to this paper.

^♯^Corresponding Author:

Dr Sandrine Thuret, Department of Basic and Clinical Neuroscience, Institute of Psychiatry, Psychology and Neuroscience, King’s College London, 125 Coldharbour Lane, London, SE5 9NU, UK

Telephone number: +44 (0)20 7848 5405

Email: sandrine.1.thuret@kcl.ac.uk

# Affiliations

^1^King’s College London, Social, Genetic and Developmental Psychiatry, Institute of Psychiatry, Psychology and Neuroscience, London, United Kingdom.

^2^National Institute for Health Research Biomedical Research Centre for Mental Health, Institute of Psychiatry, Psychology and Neuroscience at the Maudsley Hospital and King’s College London, UK.

^3^King’s College London, Department of Basic and Clinical Neuroscience, Institute of Psychiatry, Psychology and Neuroscience, London, United Kingdom.

**Supplementary Information**

**S.1: Further information on cell experiments**

RNA samples were collected from a series of cell experiments assaying the effects of antidepressants [escitalopram (0 nM, 145 nM, 290 nM, 1160 nM) or nortriptyline (0 nM, 267 nM, 534 nM, 1068 nM)]. All cells were grown as described in the main text to generate either proliferating or differentiating cell populations. Drugs were added to cells 24 hours after seeding (proliferating and differentiating conditions) and again at the beginning of the differentiation protocol (differentiating conditions only). Neither of the drugs elicited genome-wide effects on expression, but the inclusion of drug and drug dose as covariates in our analyses allowed us to regress out any minor effects on gene expression.

**S.2: Representative images of human hippocampal progenitor cells during proliferation and neuronal differentiation.**

**
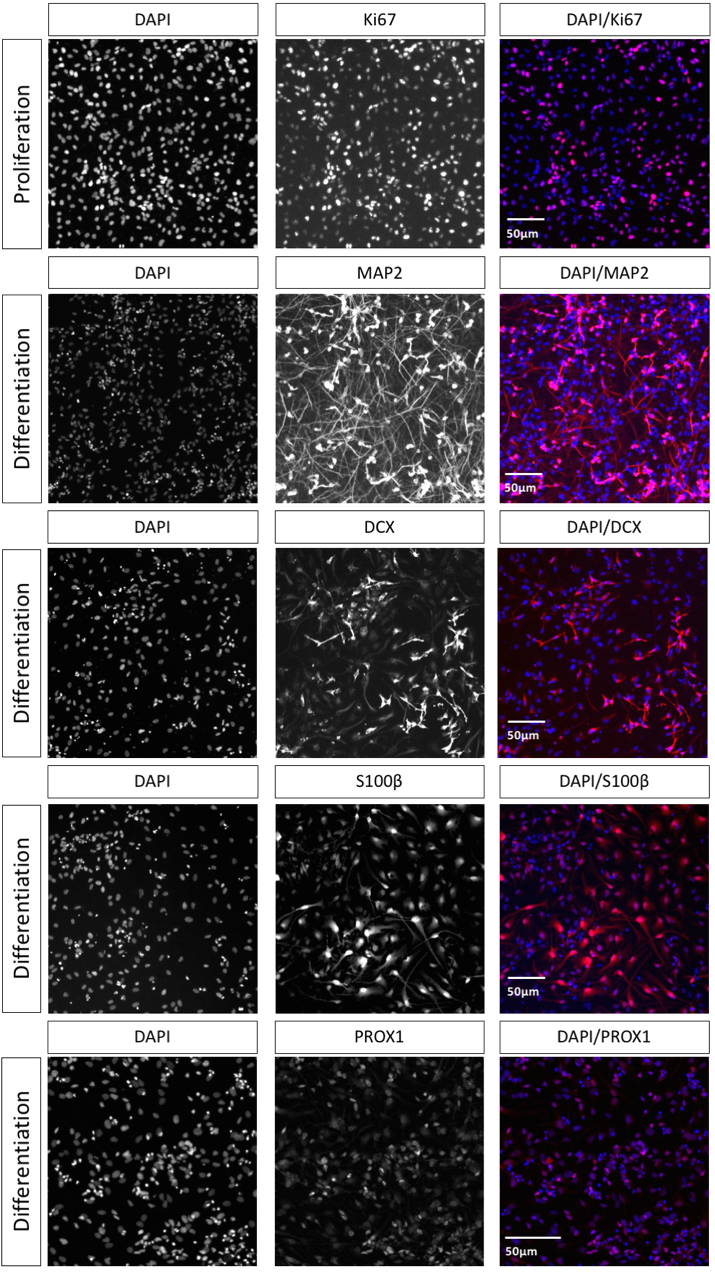
**

S.2: Proliferating human hippocampal progenitor cells as labelled by Ki67 (red). Human hippocampal progenitor cell-derived neuroblasts labelled by Doublecortin (DCX) (red) and neurons detected with Map2 (red). Human hippocampal progenitor cell-derived astrocytes are detected with S100β staining (red). Differentiating hippocampal progenitor cells also express a dentate gyrus specific neuronal marker, Prox1 (red). Nuclear staining DAPI is in blue. Staining was performed as described in main text.

**S.3: Primer Sequences**

S.3: Primer sequences used for qPCR validation experiments.

| **Transcript** | **Forward Primer (5'->3')** | **Reverse Primer (5'-> 3')** |
| --- | --- | --- |
| TGFBI | ACTTGGATGTCACTGCCTGA | AGCCCCACAATACTGAAGCT |
| BCAN | CTGCCCTGTCACCCAAATTT | GGTATTTCCCAGAAGCCCCT |
| VIM | CTTTGCCGTTGAAGCTGCTA | ACGAGCCATTTCCTCCTTCA |
| DCX | GCACCTTACACACATGGCAA | CCCCTCCGCAATTCAATGAG |
| CDC20 | GCTATGGCGCTGTTTTGAGT | GTGGATGAGGCTGCTTTTGG |

**S4: Immunocytochemistry**

For a subset of differentiating cells (n=17) we had concurrent staining data which allowed us to estimate which cell types most likely drive expression changes during differentiation.

Differentiating cells were grown concurrently in triplicate in 96-well plates for 17 differentiating cell populations, matched to those grown in 6-well plates for the gene expression experiments (passage, media, conditions were the same). Cells were grown as described above, but at the end of the protocol, media was apirated and cells were fixed in 4% paraformaldehyde for 20 min at room temperature, and then stored in phosphate buffered saline at 4°C. PBS was then aspirated and 50 μL of blocking solution (0.3% Triton X-100, 5% normal donkey serum (Alpha Diagnostics, San Antonio, TX, USA) in PBS) was added for 1 hour and left at room temperature. Primary antibodies were subsequently diluted in blocking solution and added in 30 μL per well and left overnight at 4°C. Differentiation was assessed using doublecortin (Dcx), which is present in immature neuroblasts (Rabbit; anti-Dcx; 1:500; Abcam), and microtubulin-associated protein-2 (MAP2) which is present in more mature neurons (Mouse; anti-Map2; 1:500; Abcam). Gliogenesis was characterised using S100 calcium-binding protein β (S100β) in differentiating cells (Rabbit; anti-S100β; 1:500; Dako, Glostrup, Denmark).

The following day, primary antibody solutions were removed and washed twice with PBS. Wells were then incubated for 30 minutes at room temperature in blocking solution. Following this, secondary antibodies diluted in 30 μL of blocking solution were added and allowed to incubate at room temperature for 2 hours. Secondary antibodies used were all manufactured by Invitrogen and were diluted in 1:500. Secondary antibodies include: Alexa 555 goat anti-rabbit and Alexa 555 goat anti-mouse. Wells were then washed twice with PBS and 50 μL 4',6-diamidino-2-phenylindole (DAPI; 1:2000; Sigma) was added per well (for nuclear staining). Wells were then washed three times with PBS and stored in a final third wash at 4°C in preparation for immunofluorescence detection. The CellInsight NXT High Content Screening (HCS) Platform (ThermoScientific, Wilmington, DE, USA) was used for immunofluorescence detection. This allowed for an unbiased quantification of the percentage of cells expressing each of the three markers. 15 independent fields were assayed per well and the inclusions of negative staining controls allowed for fluorescence background correction. We subsequently used the average percentage marker expression from all fields within our three technical replicates in downstream analyses.


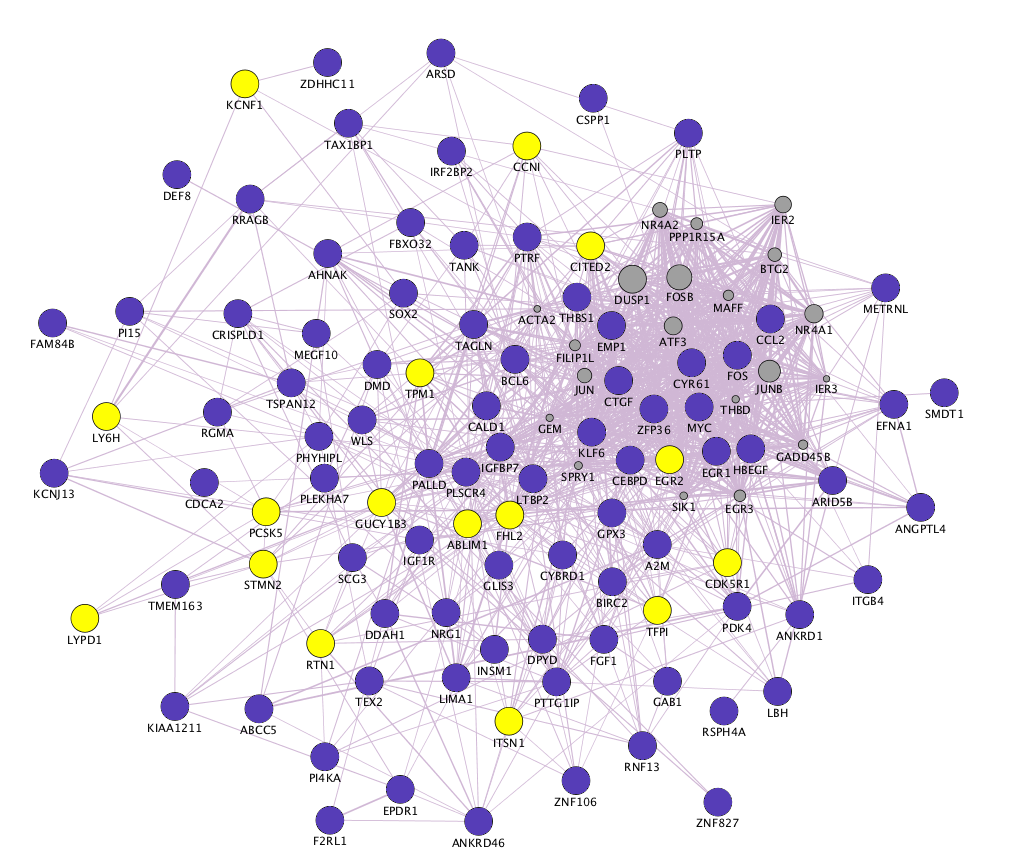


**S5: A gene co-expression network consisting of upregulated transcripts associated with the generation of doublecortin-positive neuroblasts.** Genes shown in purple represent transcripts upregulated during the generation of human doublecortin-positive neuroblasts. Genes shown in yellow represent transcripts upregulated in human doublecortin-positive neuroblasts, and which are also overexpressed in mouse neurons relative to other central nervous system cell types. Genes shown in grey represent hypothetical transcripts output from GeneMania.


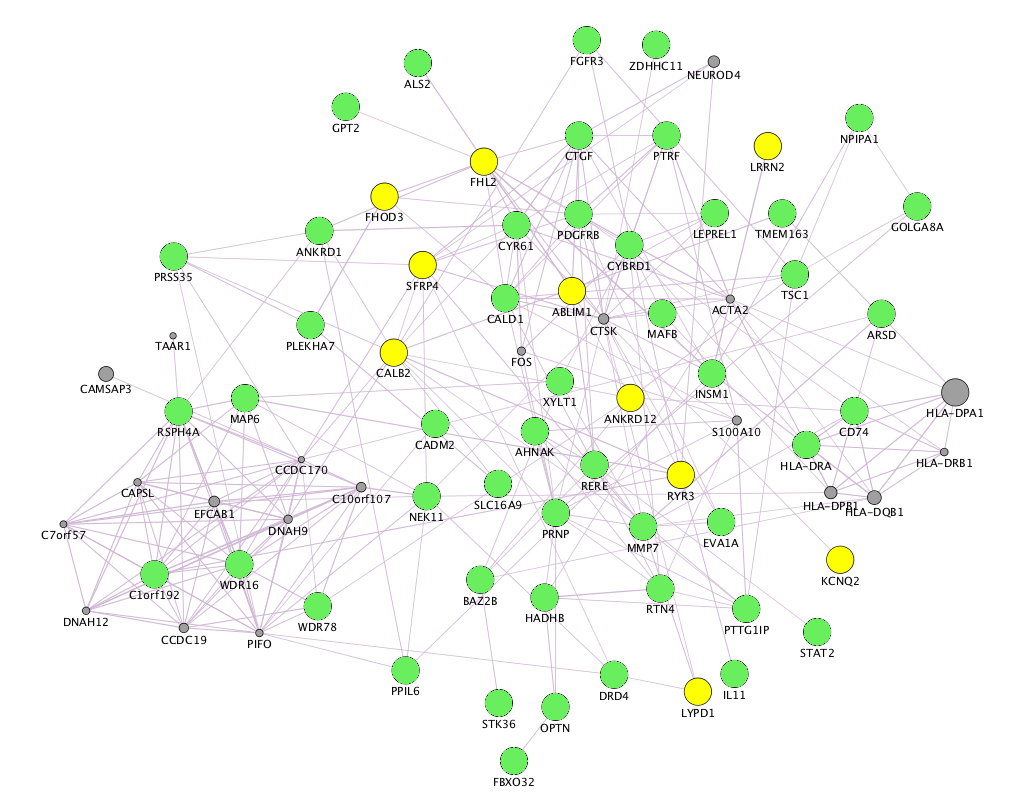


**S6: A gene co-expression network consisting of upregulated transcripts associated with the generation of MAP2-positive neurons.** Genes shown in green represent transcripts upregulated during the maturation of neurons. Genes shown in yellow represent transcripts upregulated in MAP2-positive neurons, and which are also overexpressed in mouse neurons relative to other central nervous system cell types. Genes shown in grey represent hypothetical transcripts output from GeneMania.


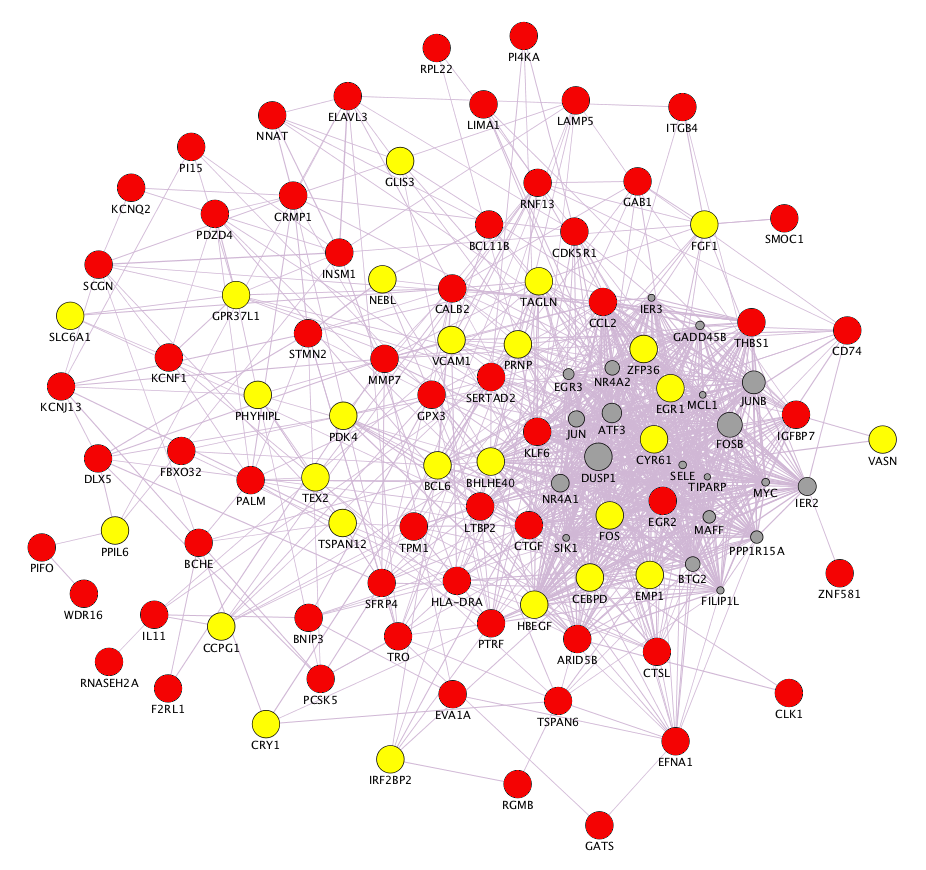


**S7: A gene co-expression network consisting of upregulated transcripts associated with the generation of S100β-positive astrocytes.** Genes shown in red represent transcripts upregulated during the generation of human S100β-positive astrocytes. Genes shown in yellow represent transcripts upregulated in human S100β-positive astrocytes, and which are also overexpressed in mouse astrocytes relative to other central nervous system cell types. Genes shown in grey represent hypothetical transcripts output from GeneMania.

**S8: Interpreting cell-type-associated analyses and comparison to mouse**

As a secondary consideration, we also linked our gene expression data with corresponding immunocytochemistry data which was available for a subset of our sample, which allowed us to estimate cell-type-associated expression changes during progenitor differentiation. These cell-type-associated transcripts included genes previously found to be enriched in either neurons or astrocytes in mouse. Such as using neuronal and astrocytic cellular mice transcriptome databases, we found that 20% of the transcripts upregulated in human neuroblasts/neurons networks (S.5-S.6) are also overexpressed in mouse neurons relative to other central nervous system cell types and 50% of the transcripts upregulated in human S100β-positive astrocytes are also overexpressed in mouse astrocytes (S.7), suggesting that there are at least some comparable changes to expression profiles across both mouse and humans during hippocampal brain development and future studies should explore further in details the meaning and developmental timing of these overlaps and differences.

**S9: ENIGMA-VIS (http://enigma.ini.usc.edu/enigma-vis/) results showing single nucleotide polymorphisms (SNPs) within (a) *CEPBD*, (b) *EGR1* and (c) *EGR2* and their association with adult hippocampal volume.** Points represent individual SNPs within each of the genes, with the radius of each circle corresponding to the minor allele frequency for that SNP. The x-axis corresponds to base-pair/chromosomal location, and the y-axis represents the associated –log10(p-value) generated from the ENIGMA GWAS.

(a)


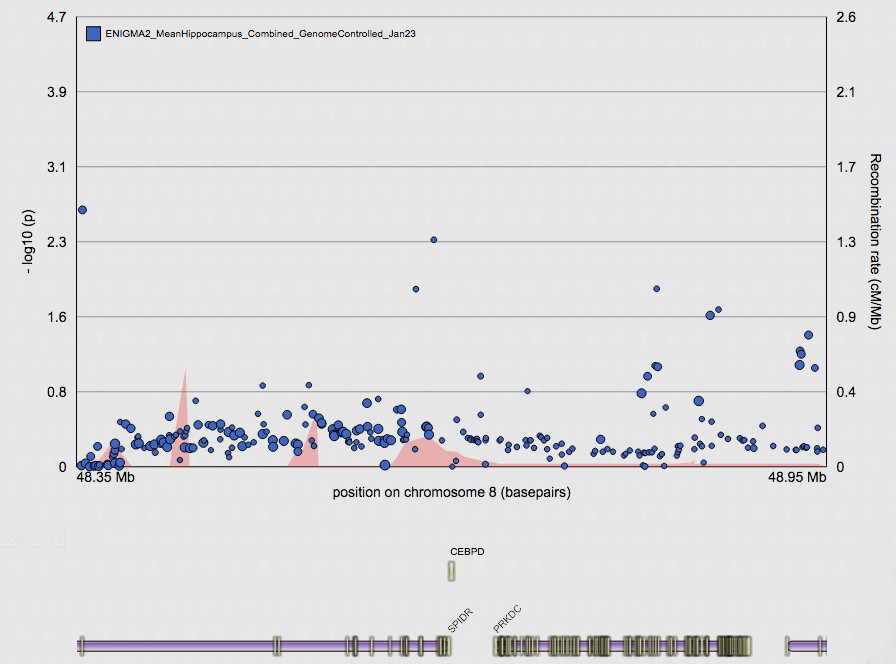


(b)


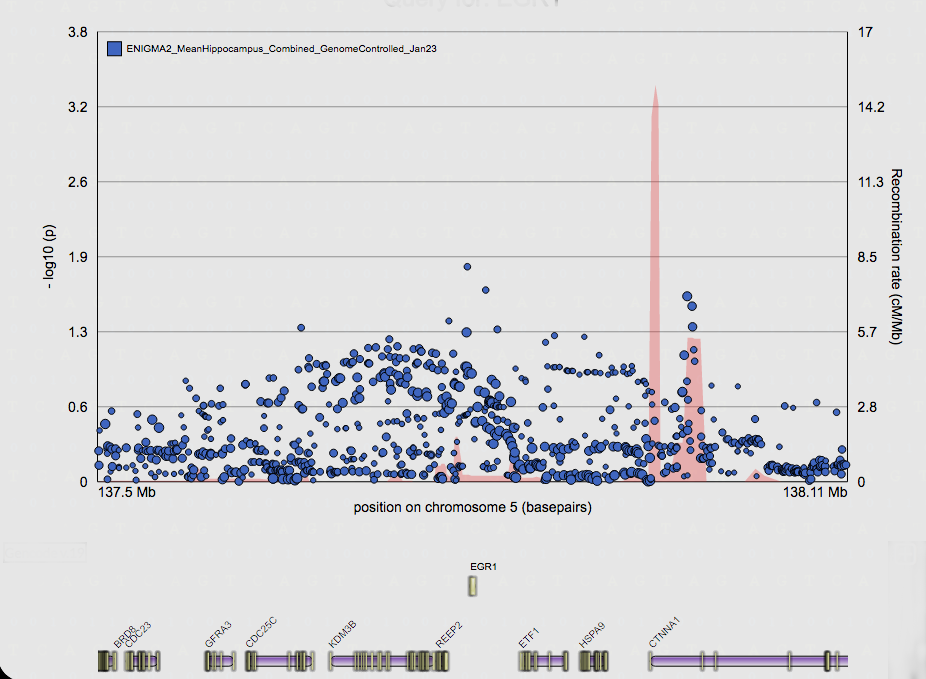


(c)


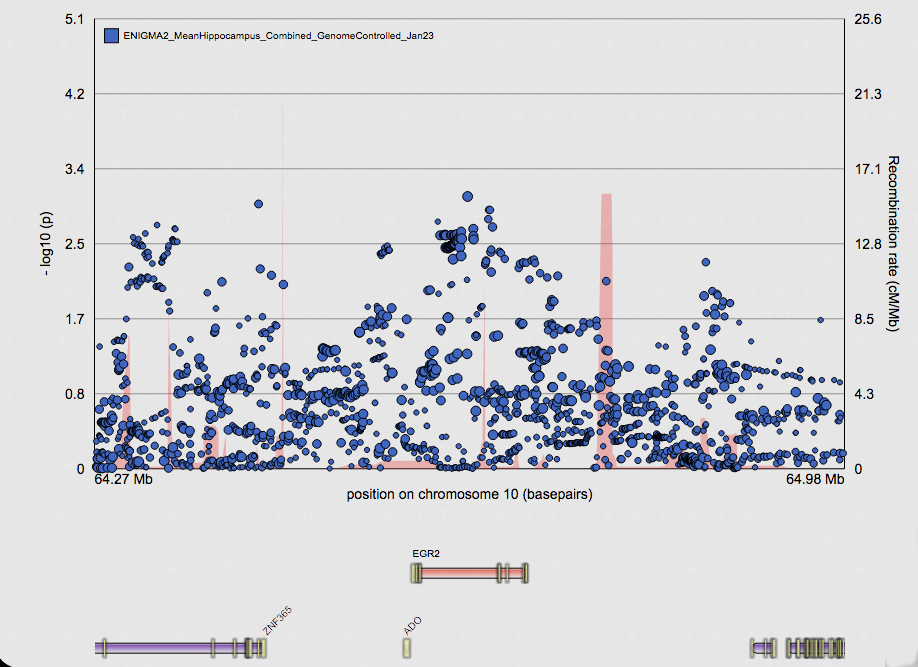


rs7913336
